# Extracts

**Published:** 1882-05

**Authors:** 


### Extracts.

---

Oophorectomy.—The subject of oophorectomy has now for a considerable time been before the profession, and, although it was so fully and fairly discussed at the recent International Congress in London, it will still require a long time before many points in connection with it, which are now *sub judice*, can be satisfactorily solved.

The three main points to consider are, its safety, its facility, and the selection of the cases suitable for its performance. First, its safety can now be said to be established. Without being able to draw a line above which its mortality would be considered high, and at or below which it would be considered normal, I do think the maximum mortality ought not to exceed 5 per cent. Dr. Robert Battey recently prepared a table of all the cases that he could obtain information of, and he found the mortality to be about 18 per cent. In my practice I have had forty complete cases, with a result that all have recovered from the operation, and I believe that nearly every one has been cured of the disorder for which the operation was undertaken.

Since it is a well established fact that increased experience in abdominal operations, perhaps more than any other branch of surgery, tends to increased success, there will always be, as it were, two kinds of mortality—a heavier mortality, the result of a general statistical record, including therein all operators, the less experienced and the experienced; and the lighter mortality of those surgeons who have had considerable experience, and who have been enabled by their special skill to reduce the death rate to its lowest limit.

Secondly, the facility of oophorectomy is a point requiring a few words. Some operators consider that it is both more difficult and more dangerous than ovariotomy. I, myself, am of the opposite opinion; although, exceptionally, as all know, nothing may be more easy of performance than a certain case of ovariotomy.

As regards the special details of the operation itself, three different methods of reaching the ovaries have been recommended. (*a.*) The vaginal incision. The earlier cases were generally performed by this method, and most probably the high mortality spoken of above may be largely due to it. When we consider the unavoidable difficulties, and the liability to septic danger connected with this method, and also the ease of the abdominal method, the wonder is that it has not been discarded altogether. It is still practiced in America, and I believe also to some extent on the Continent. There is no case that could be done by the vaginal method that could not be more easily and safely done through the abdominal wall; and, certainly, ovaries that could not be removed by the abdominal incision, where everything is open to view and space is abundant, could not be removed through the vaginal roof where space for manipulation is necessarily very limited, and the sense of touch is alone our guide. In addition, in opening the vaginal roof there is a great danger of wounding the intestine.

(*b.*) The direct lateral incision, *i. e.*, an incision on each side of the abdominal wall over the corresponding ovary. This is not now practiced. It is more dangerous and troublesome than (*c*), the median incision, which is the one usually practiced as being safe and simple.

It has been advised of late that the tubes should be removed in all cases where practicable, as a step more likely to secure the results desired from the operation than if they are left behind. I take it that the additional benefit of removing the tubes over and above that of only removing the ovary is, that it involves inserting the ligature more deeply down in the broad ligament, and so affecting the supply of blood to the uterus. Mr. Spencer Wells has recently endeavored to show that this result is obtained. I think it must be by the effect which is produced by the ligature of the ovarian artery: the uterine artery would be affected only indirectly, through its anastamosis, as it would be too far away to be caught by the ligature.

Thirdly, as to tho selection of cases suitabie for oophorectomy. Here we are confronted with considerable difficulty. In fact this is the point upon which there is the principal dispute and criticism between the advocates and the opponents of the operation. The combined experience of those who have been engaged in this special work has not, up to the present, been able to define very exactly the limit and extent of its usefulness. Since it is an operation of election, time must elapse and results must be known, before we shall be able to say exactly what class of cases should, and what should not, be subjected to it. Dr. Robert Battey, the originator of the operation, and by whose name it is sometimes called, gives a safe rule which is embodied in the three questions :—is this a grave case ? Is it incurable by any of the resources of the art short of the change of life ? Is it curable by the change of life ? If all three of the questions can be answered affirmatively, the case is a proper one. If not, the operation is not to be justified. The question of the occurrence of menstruation after the removal of the ovaries is not yet quite cleared up. A few cases have been met with where it has continued after the operation : I believe that these are to be looked upon as quite exceptional, and that beyond the slight metrotaxis shortly after the operation, the catamenia do not reappear. This is providing the tubes and the whole of the ovaries be removed. It may appear strange even to raise a doubt as to the whole of the ovary not being removed, but the mesovarium is sometimes so short as to be with difficulty ligatured, except deeply down in the abdominal cavity, and, when a ligature is applied, there is found to be so very little tissue on the distal side, that it is easy in one's anxiety to leave enough for safety, the knife or scissors may encroach on ovarian tissue ; and when this happens the operation is *pro tanto* a failure, and that it does occur sometimes is I think likely. This being so it must affect the secondary result of the operation. I am very sorry that I have not preserved more accurate notes of my earlier cases, in reference to the question of subsequent menstruation. In them I was in the habit of removing the ovary alone, or the ovary and tube together, according as it was easier, or appeared more convenient, and I have met with a few cases of the series where menstruation afterwards has been observed : but I am not able to identify them sufficiently for scientific accuracy. Of late, I have endeavored, in every case, to remove both ovary and tube, both to avoid menstruation and for more completely effecting the object in view for which the operation was undertaken. I have not seen, in one of these cases, any subsequent menstruation. It is therefore, only an impression, but a strong one, made upon my mind, that if both tube and ovary are removed, no menstruation will follow, but that it may be expected to do so if the ovary alone is removed, the tube being left behind. —*Thomas Savage, M.D., M.R.C.P., Lond., in Birmingham Med. Review.*

---

THE EARLY TREATMENT OF PROSTATIC OBSTRUCTION.—It may be generally stated that of males who have passed fifty-five years of age, about one-third sooner or later have enlargement of the prostate ; of these about one-half suffer therefrom, though so long as micturition is efficiently and painlessly performed, there are seldom grounds for complaint.

It is exceedingly interesting, as indicating how relief may be afforded, to analyze the cases where the prostate is large but does not obstruct ; there are at least two conditions explanatory of non-interference with micturition under these circumstances.

First, where the hypertrophy is towards the rectum and the relations of the prostatic urethra are not altered ; and second, where the hypertrophied gland is lobulated and channels are left between the masses along which urine flows without interruption. A careful consideration of these conditions has suggested that they are capable of artificial production to a useful extent.

The teaching of the present day is, however, to the effect that mechanical treatment is not to be employed until either retention occurs or the bladder becomes inflamed, then such means may be resorted to. It was asked why the same treatment should not be applied as in the case of urethral stricture. The objection generally advanced is that irritation will be produced. It was urged that there was no evidence in support of this objection. On the contrary, the prostate was about the most long-suffering organ in the body, and though it was subjected to a great variety of mechanical expedients, in lithotomy and other operations, it rarely became inflamed. It might just as well be said that because strictures were found occasionally to be exceedingly irritable, treatment was to be postponed until retention of urine or cystitis were provoked. But if intolerance to early mechanical treatment were proved, it was only postponing the day until the necessity was greater and the difficulty more apparent. If there was danger of irritating the prostate, it was none the less because its size was larger.

With the view of obtaining similar conditions to those occurring naturally in large prostates, where there is no interference with micturition, a mode of treatment with specially-adapted bougies was described. The instruments are gum-elastic, two to four inches longer in the stem than usual, with an expanded portion an inch from the tip, which is made to enter the bladder. In this way the prostatic urethra is subjected to pressure on the insertion and withdrawal of the instrument.

As a rule, if dilatation is not too rapidly proceeded with, no irritation is aroused. On the contrary, greater toleration of urine follows, owing to the ease and completeness with which the bladder is then emptied.

In a few persons it became necessary to establish a state of instrumental toleration, the frequency for doing this, as a rule, depending more on the manipulator than on the instrument. In some individuals intolerance of urethral interference was entirely due to the condition of the urine. Such sensitiveness had been traced to the presence of uric acid in unnatural quantities and form. On the correction of this, patients previously intolerant of instruments were found capable of undergoing the necessary mechanical treatment with the greatest advantage.

In advocating the early treatment of prostatic obstruction by the means referred to, the author had already had sufficient proof of its efficacy. He had demonstrated that the regular use of the dilators was capable of so moulding the enlargement as to prevent obstruction. Cases were under observation where the symptoms indicated that an impediment to micturition was commencing to form. Such patients in this way regained the power they were beginning to lose. In bringing forward his views on the subject, he did so with the feeling that little had been yet done towards preventing the progressive development of a condition which was often followed by very distressing, and sometimes embarrassing, results—results which we knew of and stood by to palliate, though we had hitherto been helpless in preventing them.—*Reginald Harrison, F.R.C.S., in Med. Press and Circular.*

---

THE RELATION OF SYPHILIS TO SCROFULA.—Let us study the affirmative side of the question.

I. Syphilis and scrofula undoubtedly show a tendency to affect similar structures, as is proved by the many diseases, known and admitted to be inherited syphilis. In fact, those who hold that the two affections are distinct are obliged to admit that it is sometimes impossible to determine, from symptoms, or even from the antecedent history obtainable, which disease is responsible for the lesions exhibited.

II. Scrofula has been, and is, most common at times of life, in historic periods, and in countries in which syphilis is known to have been and to be prevalent and virulent. Again, scrofulous diseases are less prominent and more benign at the present day than formerly, when acquired syphilis was more destructive, because less judiciously treated.

III. It is so well known that syphilitic parents have, as a rule, syphilitic children, that an exception gives rise to scientific comment. Scrofula is usually an inherited condition, and could readily, therefore, be due to such a wide-spread and prolific parentage as syphilis.

IV. Scrofulous affections are found in the highest stations of life—a fact which would be well-nigh inexplicable if scrofula were the resultant of exposure, insufficient food, filth and bad air. If it be a grandchild of syphilis, however, its presence in every household can be as general as the immorality that gives birth to its ancestor.

V. Cases occur similar to that reported by Lugol, who refers to a family of two healthy children, the father of whom subsequently contracted syphilis and inoculated his wife. A third child was born, who, after exhibiting many scrofulous symptoms, died at eighteen years, though the elder brother and sister presented no such conditions of ill health.

VI. Acquired syphilis has been accused of awakening scrofulous manifestations in those predisposed to scrofula, in whom no symptoms have occurred for years. This suggests a relationship between the affections which is very intelligible if scrofula be viewed as a remotely inherited syphilis.

VII. Scrofula is the condition furnishing deposits of tubercle, which are the result of infection arising especially from cheesy accumulations. Syphilis is an incessant instigator of inflammations and of gummy tumors which frequently undergo caseous degeneration. Hence, pathology points at syphilis as an agent well calculated to give birth to the scrofulous diathesis.

VIII. The most effectual remedies in the treatment of syphilis, whether acquired or inherited, are mercurials and preparations containing iodine. These so called specifics in the management of syphilis are agents of greatest benefit in alleviating scrofulous manifestations.

The arguments of those denying the syphilitic parentage of scrofula may be summarized as follows:

I. Scrofula is not inoculable, as are the primary, the secondary, and sometimes the inherited lesions of syphilis.

II. The successive steps between primary syphilis in the progenitor and scrofulous diseases in the posterity have not been traced by any scientific investigators; who quote only isolated cases of apparent relationship.

III. Scrofulous affections are found in patients who have presented no such manifestations in early life (when inherited syphilis is especially noticeable), and who have never acquired primary syphilis. Moreover, phthisis, which is a lesion of scrofula, is found very frequently in animals not known to have syphilis.

IV. Scrofula is not considered a factor in the causation of syphilis, as might be expected if there was a mutual relationship between them.

V. Scrofula is believed by some to have existed in Europe before the advent of syphilis, and according to certain writers is now prevalent in countries where syphilis is rare.

VI. Syphilis yields more quickly and completely to treatment than scrofula, though the same or similar remedies may be available in both diseases.—*John B. Roberts, M.D., in Philadelphia Medical Times.*

---

Billroth's Operations.—It is no wonder that Billroth does remarkable operations. In the first place, he is responsible to no one; there is nobody to question him and to ask, why do you do this, or why do that? The patient has not a word to say in the matter. If Billroth determines to do an operation, that is the end of it; he is supreme. If the patient recovers, all right; if he dies, all right; not a particle of difference either way. I do not know if he even has any particular satisfaction in the recovery of the patient; it all lies in the fact of having done the operation. In the second place, Billroth has been first professor for years. He has the most abundant material for all classes, qualities and kinds. He does all kinds of surgery, including everything relating to the female generative tract. There is no specialty of gynæcology of any consequence here. There is not a day in the year, and has not been for years, that Billroth has not done major operations. I do not mean amputations of limbs or resection of joints—he would not look at such a thing. Why! he whips out a goitre as a sort of by-play while the patient is being etherized. To take out a tongue is easy for him, and he ties the lingual arteries on both sides with the utmost ease. So exceedingly familiar is he with the topographical anatomy of the body that he rarely uses a director, but cuts right down to the place. He stops at nothing. The other day he was removing a cancerous ovary which was found to be adherent to the bladder and part of the small intestine. Does he stop? No! He cuts out a section of the bladder, stitches it up, cuts off seven inches of the intestine, stitches the ends together, removes the growth, closes the wound, and the woman recovers.

I saw a man in the ward with a cancer of the stomach at the pyloric end, and after opening the abdomen, he found the disease so extensive, involving so much, that he could not remove the growth at all. Does he close up the wound? Not he! He cuts down to the healthy gut, snips it off, cuts a hole into the healthy part of the stomach, stitches the gut to it, and the man is getting fat. Now, I say that, to be sure, they are wonderful operations; but why shouldn't they be? Billroth has attained this boldness and amazing skill in surgery by easy stages and after years of daily operating. Another thing, if he proposes doing an operation a little new or out of the way, he has one cadaver or a dozen to experiment upon, if he wants them, at any time or hour of the day. There are twenty to thirty bodies in the pathological rooms every morning.

In Heitler's ward I was shown a case of pleurisy with large effusion. When I asked for treatment, he said the patient will get no medicine. And though the chest was bulged out enormously, he would not tap, because he said it was bad practice. The patient did well. They give no medicine for pneumonia except "maybe a little *ipecac.*" You would be amazed at the number of doctors I meet here who are skeptical as to the efficacy of medicine.

Volkmann of Halle, one of the very remarkable joint surgeons of the continent, cuts right plumb into a joint, knee, hip, or elbow, and has good results.

The great antiseptic is iodoform. It is used in every operation and every character of sore. After the operation is over the wound is covered with fine mosquito netting, dusted full of iodoform, so that it looks like yellow mosquito bar; then absorbent cotton is next applied, following this, cloths wet with carbolized water, and over all the roller bandage. When operations for a cancerous vagina, os, etc., are done, the vagina is stuffed with iodoform tampons, and the wound not looked at for days. When the dressings are removed they are sweet and pure. Old cancerous sores are dusted over with iodoform. One day I saw an assistant blowing iodoform into a cancerous mouth. There was not a particle of smell from it, although hitherto the ward was made unbearable with it.—*McClelland in Medical Times.*

---

THE VICTIMS OF THE RING THEATRE, IN VIENNA.—The *Allgemeine Medicinische Central-Zeitung* publishes Professor Hoffman's report on the methods adopted for the identification of the victims of this great fire. Some of his observations will no doubt be of interest to our readers. In this report he says: Over two hundred and fifty bodies were examined, and a great many among them identified. In many cases, owing to almost complete carbonization, sex could only be ascertained by internal examination; the presence or absence of the uterus and ovaries being our only guidance. Age could likewise only be approximated, yet was of importance in establishing identity. Simple inspection often revealed strange transformations: age bore the semblance of youth, and reciprocally. In such cases we had to rely on the indications furnished by the humerus, with regard to complete union between the diaphysis and epiphysis. As you are aware, this only becomes perfect after the twenty-fourth year. In like manner, the ossification of the ribs and larynx served as indices; for you remember, with this last it commences at from thirty to thirty-five, and is complete by about the fortieth year. The condition of the ovaries supplied valuable information. In girls and young women they were quite smooth, but in older subjects they had that well-known uneven appearance which they assume after numerous ovulations.

The hair and beard on a great many bodies had become of a uniform black color, and only by careful cleansing were they restored to their natural hue.

The color of the eyes was not easily detected, the cornea having a milky and cooked-like appearance. In some cases an opalescence of the iris led us to believe the eyes were blue.

The teeth were calcined, and so brittle that they crumbled under the least touch. They looked as if carious, and it was often very difficult to judge of age from the amount of wear they presented. We found great quantities of artificial teeth.

When the hands were under inspection the condition of the epidermis and finger nails supplied indications. In proof of this, let me tell you an episode. An aged couple and their son were in search of a young woman, the wife and daughter. As an indication, they said her finger nails were red, from nursing. A body was found with breasts and nails answering this description, but around the neck was an "amulet" and cross, and the sorrowing family were Jews. Later, however, another body was discovered, and fully identified by them.

Under the head of "particular marks," we noticed a cicatrix from coxalgia, and a cyst at the root of a right index finger. Of course, we found many hernias and hernial bandages.

In all, we ascertained six cases of pregnancy, of which one was in the six month, two in the second, and two in the third. In one of these last, all that remained was the neck, the uterus and ovaries having been carbonized. The breasts contained some colostrum. This body was identified.

Surfaces still covered by portions of clothing were found well preserved, even upon bodies otherwise greatly carbonized.

As may well be imagined, we met with burns of every degree, but generally speaking, only very few bodies bore the appearance of having been burned while alive; external erythema and phlyctenulæ being quite rare. It is probable that the majority of the victims in this terrible catastrophe were first smothered by smoke, and only burned while in asphyxia, in agony, or after death.

In some cases skulls had been carbonized and split open by the heat, exposing the calcined brains; and many abdomens were found gaping open, their shriveled contents having remained undisturbed.—*Med. and Surg. Reporter.*

---

A New Sanitary Inspector.—Cats used as pointers to discover defective plumbing.

An experiment tried recently by a woman in Hoboken to detect the presence of sewer gas in her rooms was a topic of conversation among the Sanitary Inspectors at the rooms of the Board of Health yesterday. The woman had noticed an offensive odor in her parlor, and she went to the agent of the house to request that a plumber be sent to examine the drainage pipes. The agent told her the plumbing in the house was perfect. She went home and called in some neighbors, who thought sewer gas was escaping from the waste pipes. Acting on the suggestion of a friend, she sent out for some oil peppcrmint, and poured it into a stationary wazhbasin on the third floor. From the basin the oil passed down through a waste pipe behind a closet off the parlor. Very soon the odor of peppermint pervaded the parlor. The woman then went to the agent again, and told him she was convinced that there was a break in the waste pipe on the first floor of the house, at the time telling him of her experiment with oil of peppermint. The agent refused to send a plumber, declaring that the odor of peppermint was so penetrating that it would soon fill a whole building. After studying over the situation for some time, the woman purchased some oil of valerian and poured it into the wash-basin upstairs. She then borrowed from her neighbors two able-bodied cats and placed them in the parlor. The cats snuffed the air in the room as if it were agreeable to them, and they both went toward the door of the closet. When the closet door was opened for them they went in immediately and sprang upon a shelf, where they remained, purring and still manifesting unmistakable delight. The woman went to the agent's office and related what she had done. Although incredulous still, the agent sent a plumber with directions to tear away the lath and plaster in the closet at the point where the cats had rested in their hunt for the valerian. The plumber found behind the shelf the waste pipe completely disjointed. The break in the pipe was large enough to allow an unwholesome amount of sewer gas to escape in the house. Some of the Sanitary Inspectors said yesterday that the experiment was new and decidedly ingenious. They thought that the cats might be used in a similar manner in this city to more advantage than in Hoboken. By employing their household pets as pointers, it was said, residents of the city might save themselves from illness, from poisonous gases, and also save the cost of employing engineers to examine the drainage in their houses.—*Lancet and Clinic.*

---

SURGICAL USES OF KANGAROO TENDON.—A paper was read on this subject by Mr. Girdlestone, at the meeting of the Royal Medical and Chirurgical Society on February 14. The author stated his belief that for tying large vessels in their continuity, the long even tendons from the tail of the kangaroo were as stiong as the silk ligature, caused no ulceration in the coats of the vessel, and had all the valuable qualities of catgut, without its defects. The tendons also make excellent sutures, resisting the effects of purulent discharges for a long period. A reef-knot does not slip or become loose. Where there is no suppuration, the tendon coalesces with the tissues, and in suppurating wounds, though softened, they are found still to hold together at the end of eight days. The tendons were previously immersed for some weeks in carbolic oil, and entire tendons only were used. They have been employed in ovariotomy and in vesico-vaginal fistula, and held till union took place. In plastic operations—hernia and varicocele—the tissues have readily healed over them. A medium-sized tendon is so strong that it is difficult to break it with one's hands, and it is uniformly strong in its entire length of from twelve to eighteen inches. Tendons can be hardened for use as sutures by immersion in a half per cent. solution of chromic acid, after which they can be also used as drains for wounds. In their preparation they should not be removed from the tail *en masse*, but one at a time and without force, as, if split longitudinally, they cannot be relied on, and should not be used. Every diameter that is required is attainable, so that there is no occasion for splitting, neither should two or more tendons be twisted together, as it destroys their flattened form. On removal from the recently killed animal, they should be cleansed in water, and afterwards in a carbolic acid solution, and then dried; and can be so preserved, or in a five per cent. carbolic oil as most convenient, to be again steeped in a watery solution of carbolic acid before use. *Lancet—N. Y. Medical Record.*

---

State Examinations in Alabama.—I have recently read several articles in your journal on the subject of State examinations and legal requirements for those who propose beginning the practice of medicine. As you have not mentioned the laws of Alabama on this subject, I take it for granted you are unaware of them. These laws have accomplished great good during the five years of their existence, not only by returning to their colleges recent graduates who were incompetent, but in freeing the state of those great evils that travelled from state to state and imposed upon the afflicted and the credulous. "The renowned cancer doctor" will not "stop for three days only," but gives our State a wide berth. Under the laws of Alabama each County Medical Society is authorized to elect a board of five censors, whose duty it is to examine all applicants who are graduates in medicine, and who propose beginning the practice of medicine. A State board is authorized to examine all who apply, and to license when qualified, without regard to their having attended college; and no one can begin the practice of medicine in this state, under penalties of the law, without going before the County or State Board and obtaining a license.—*J. H. Gunn, M. D., in Medical Record.*

---

Arrests For Advertising Nostrums.—Through the efforts of Drs. J. N. Dickson and W. R. Hamilton, of Pittsburgh, Pennsylvania, (*Medical News*, February 4, 1882,) there have been several arrests and convictions of irregular practitioners and others for "advertising medicines, drugs, and nostrums for the cure of secret diseases," in violation of the law of 1870 of that State. Dr. C, R. Lightner (a graduate of Bellevue Hospital Medical College) was arrested and convicted for advertising (in common with his partner, Dr. Hartman,) Pe-Ru-Na, which, it is stated, will "restore sexual debility to the energy, fire, and vigor of youth." After conviction, Dr. Lightner was let off upon payment of all the costs, and voluntary publication of a card in the daily papers, promising "not to distribute pamphlets recommending nostrums, etc., for private diseases and female complaints." Another man was convicted of advertising "Ricord's Specific Pills" for gonorrhœa, gleet, etc. He was an ex-president of the College of Pharmacy, and took high ground when arrested, but for fear of prosecution made solemn promises, and published a card, stating that until recently he was ignorant of the law touching advertisements of certain patent medicines and nostrums, and that since he has been informed of the law on the subject, he has withdrawn his advertisements, and he declares his intention to comply with the law. Dr. George was arrested for a like offence, and lodged in jail in default of bail. He plead guilty, paid his fine on two charges, and promised to do better in future. It is a pity that some such law could not be passed in other States of the Union.—*Chicago Medical Review.*

---

Death from Umbilical Hemorrhage.—The following case recently occurred in the private practice of a London obstetric physician: After the birth of the child, the cord which was unusually stout, was ligatured with thread as usual, two or three inches from the umbilicus, a surgical knot being tied, and then divided about an inch from the ligature. The cord continued to pulsate, a fact to which he directed the nurse's attention. In about seven minutes the mother flooded and the child was wrapped in flannel and laid on one side. In about twenty minutes, when the flooding had been controlled, the child was looked at and found to be lying dead in a pool of blood. The ligature was still firm. The inference is that it must have been placed over a nodosity of the cord and must have slipped on to a portion of a smaller calibre, and thus became slightly loosened. This case would tend to show that to dispense with the ligature altogether, as some German writers have advocated, is a plan not without danger.—*Medical Record.*

---

A Bogus Medical College.—We have received through the kindness of Dr. Brodie, the "annual announcement of the Detroit University Medical Department," together with some local comments upon the same.

The announcement has an imposing appearance. There is a list of thirteen professors and twenty-one lecturers. The professors have the novel prefix of "Venerand," "Very Venerand," or "Right Venerand." There are chairs on almost every subject, including "pyrology," "anthropology," and "sanitive science."

It appears that in reality "the large and commodious structure" advertised as used by the college is upstairs over a grist-mill; that the president is a cancer-doctor and a graduate of Buchanan's "Institute," and that the dean makes a specialty of "electrized anti-parasitic oxygen."—*Medical Record.*

---

Is Salicylic Acid a Specific for Rheumatism?—Dr. Lewis Shafton, Physician to the Devon and Exeter Hospitals, commenting upon the value of salicylic acid in the treatment of rheumatism, is unwilling to admit its specific properties. But Dr. Wm. Strange, of the Worcester General Infirmary, writing in the same issue of the journal after an extended experience with this drug, is confident in his statements that in the same sense as quinine acts as a specific in malaria, or mercury and iodide of potash in syphilis, so salicylic acid, but more especially its compound, the salicylate of soda, acts as a specific in acute articular rheumatism, by neutralizing the poisonous elements of the blood.—*British Medical Journal.*—*Medical Record.*

---

Medical Fees in London.—I believe that it is now the habit of the principal London physicians to charge three guineas ($15) for a visit at the house of a patient, two guineas ($10) for the first visit of the patient to the physician's office, and one guinea ($5) for a subsequent visit there. After all, a man who is believed to have special talents for healing is right to charge highly for it. The abuse seems to me to be this: whereas, any physician may charge more than a guinea, no physician is allowed by the etiquette of the profession to charge less, and yet probably there are many clever young physicians who now have very little practice, and would themselves gain and benefit others were they allowed to charge half a guinea.—*London Truth.—Lancet and Clinic.*

---

Iodoform Insanity.—According to Max Schede (Centralblatt fur Chirurgie, No. 3, 1882,) the use of iodoform externally, particularly in children, has been attended by marked psychical symptoms even at times amounting to true insanity. General mental confusion has in at least two instances been traced to it, recovering when local applications of iodoform to wounds have been removed, and reappearing on their re-application. He had also one case of deep melancholia result from its use; two cases of raptus melancholicus and three cases of simple depression. It is probable that iodoform only has these effects in patients of a neuropathic diathesis.—*Medical Review.*

---

The Late Dr. Pancoast, of Philadelphia, was one of the most opulent of American physicians. His estate is valued at about one million dollars. It is said that Dr. Gross—another eminent doctor—and he used frequently to play checkers until late hours, and that no other diversion pleased him so much. He was of old Quaker stock, and through life preserved many of the quaint traits of that people, of whom the witty Sidney Smith said that he "did not believe there ever was a Quaker baby; that is to say, one born with a broad brim and in full quake."—*Harper's Weekly.*

---

Too Much Originality and Fortune.—Dr. S. V. Clevenger, in a recent communication, makes the following citation from John Aubrey's "Lives of Eminent Persons:" "I have heard Dr. Harvey say that after his booke of the circulation of the blood came out, he fell mightily in his practice; 't was believed by the vulgar that he was cracked and all the physitians were agaynst his opinions and enveyed him." Aubrey was at Harvey's funeral, and "helpt carry him into the vault."—*Chicago Medical Review.*

